# A Pseudo-tRNA Modulates Antibiotic Resistance in *Bacillus cereus*

**DOI:** 10.1371/journal.pone.0041248

**Published:** 2012-07-18

**Authors:** Theresa E. Rogers, Sandro F. Ataide, Kiley Dare, Assaf Katz, Stephanie Seveau, Hervé Roy, Michael Ibba

**Affiliations:** 1 Department of Microbiology, Ohio State University, Columbus, Ohio, United States of America; 2 Burnett School of Biomedical Sciences, University of Central Florida, Orlando, Florida, United States of America; 3 Ohio State Biochemistry Program, Ohio State University, Columbus, Ohio, United States of America; 4 Center for RNA Biology, Ohio State University, Columbus, Ohio, United States of America; Max-Planck-Institute for Terrestrial Microbiology, Germany

## Abstract

Bacterial genomic islands are often flanked by tRNA genes, which act as sites for the integration of foreign DNA into the host chromosome. For example, *Bacillus cereus* ATCC14579 contains a pathogenicity island flanked by a predicted pseudo-tRNA, tRNA^Other^, which does not function in translation. Deletion of tRNA^Other^ led to significant changes in cell wall morphology and antibiotic resistance and was accompanied by changes in the expression of numerous genes involved in oxidative stress responses, several of which contain significant complementarities to sequences surrounding tRNA^Other^. This suggested that tRNA^Other^ might be expressed as part of a larger RNA, and RACE analysis subsequently confirmed the existence of several RNA species that significantly extend both the 3′ and 5′-ends of tRNA^Other^. tRNA^Other^ expression levels were found to be responsive to changes in extracellular iron concentration, consistent with the presence of three putative ferric uptake regulator (Fur) binding sites in the 5′ leader region of one of these larger RNAs. Taken together with previous data, this study now suggests that tRNA^Other^ may function by providing a tRNA-like structural element within a larger regulatory RNA. These findings illustrate that while integration of genomic islands often leaves tRNA genes intact and functional, in other instances inactivation may generate tRNA-like elements that are then recruited to other functions in the cell.

## Introduction

tRNAs are essential for accurate translation of the genetic code, during which the anticodon of an aminoacylated tRNA is paired with the corresponding mRNA codon. Genes encoding tRNAs that function within translation can be predicted with various software programs such as tRNAscan-SE, ARAGORN and TFAM, which classify tRNAs by their anticodon sequence, predicted secondary structure, and additional identity elements [1]–[3]. These bioinformatics approaches have led to the identification of large families of tRNAs, which in many organisms exceed the expected needs for translation alone. Recent studies have shown that canonical tRNAs can function in numerous processes outside translation, including antibiotic and cell wall biosynthesis, N-terminal tagging of proteins by the aa-tRNA protein transferases, and regulation of gene expression (reviewed in [4], [5]). The range of possible functions for canonical tRNAs has been extended by the finding that they can serve as precursors for an abundant class of stable small RNAs, tRFs (tRNA-derived RNA fragments, [6], [7]) and the observation that the corresponding genes serve as sites for the insertion of genomic islands in bacterial chromosomes [8]. Canonical tRNA structures have also been found within other RNAs, such as bacterial tmRNAs and plant viral RNAs, where they facilitate functionally important 3′-aminoacylation [9], [10]. In other instances, canonical tRNA structures do not mark larger RNAs for aminoacylation but instead provide protein-binding sites that impart a broad range of functions [11]–[13].

In addition to canonical tRNAs, a number of unusual variants have been identified that lack conserved structural features. While some of these non-canonical tRNA structures have canonical roles, for example certain mitochondrial forms lack D- and T-arms [14], others do not function in translation and are classified as pseudo-tRNAs. These pseudo-tRNAs may be tRNA relics that now maintain a different function, for example in biosynthesis of cell walls or antibiotics, regulation of gene expression, or genome replication. While a function outside translation has been defined for some pseudo-tRNAs, most notably in peptidoglycan biosynthesis [15], [16], in general little is known about the possible roles of such molecules. For example, in *Bacillus cereus* strain ATCC14597 a pathogenicity island flanks a pseudo-tRNA (tRNA^Other^, also found in some other *Bacilli*) that is only poorly aminoacylated *in vitro* and does not associate with polysomes *in vivo*, suggesting that it plays a role outside translation [17], [18]. Since tRNA^Other^ contains a Trp anticodon, its function as a regulator in the T box transcription termination system was tested, revealing a role in regulation of *trpS1* gene expression in stationary phase [18], [19]. Deletion of tRNA^Other^ is not deleterious and does not significantly change growth rates in rich media or the ability to sporulate or germinate, but does cause the cell to lose resistance to certain antibiotics. We now show that tRNA^Other^ may form a tRNA-like element within a larger regulatory RNA, consistent with previous data indicating a function outside translation.

## Results

### tRNA^Other^–dependent antibiotic resistance in *B. cereus*

**Figure 1:**
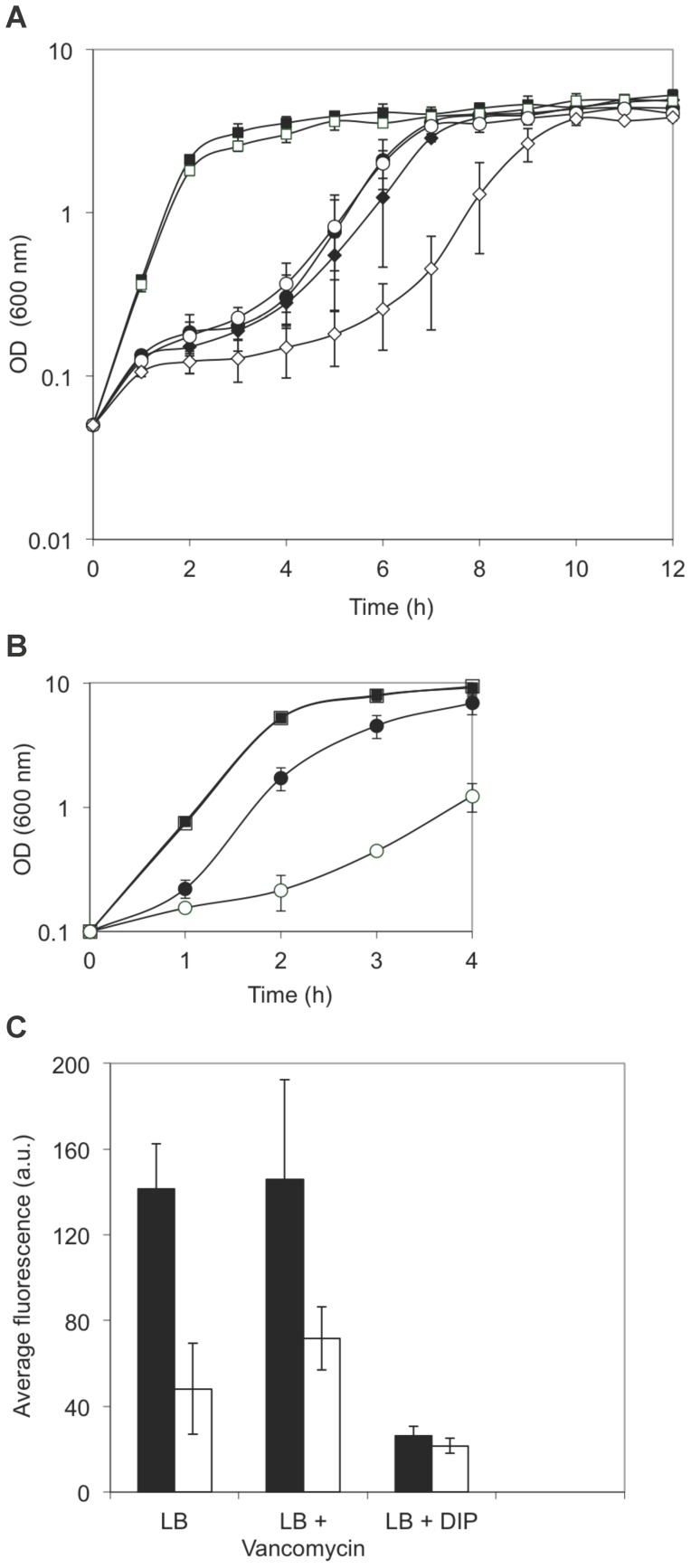
Deletion of tRNA^Other^ alters resistance to vancomycin and hydrogen peroxide in *B. cereus*. **A**, effect of tRNA^Other^ on vancomycin resistance. Wt (black) and Δ*tRNA^Other^* (white) *B. cereus* strains were grown in LB + DIP (□), LB + vancomycin (⋄), or LB + vancomycin + DIP (○) at 37°C with shaking at 250 rpm. Averages of three growth curves are shown, and error bars represent standard deviations. **B**, effect of tRNA^Other^ on hydrogen peroxide resistance. Wt (black) and Δ*tRNA^Other^* (white) *B. cereus* strains were grown in LB (□) or LB +400 μM H_2_O_2_ (○) at 37°C with shaking at 250 rpm. **C**, effect of tRNA^Other^ on nitric oxide production. Comparison of NO levels in wt (black bars) and Δ*tRNA^Other^* (white bars) cells in the presence of vancomycin or DIP. Average fluorescence is presented in arbitrary units (a.u.). When used, vancomycin was added at a final concentration of 2 μg/ml.

To investigate the possible role of tRNA^Other^, BIOLOG phenotypic microarray analyses were used to compare the growth of wt and *B. cereus* Δ*tRNA^Other^* under 1920 different conditions, of which 17 showed significant differences between the two strains (Table 1). *B. cereus* Δ*tRNA^Other^* was more sensitive than wt to a number of compounds including antibiotics, cationic detergents and positively charged ionophores. The role of tRNA^Other^ in vancomycin resistance, mechanisms of which have been extensively studied in other systems, was further characterized. At 10 μg/ml vancomycin wt *B. cereus* grew but the Δ*tRNA^Other^* mutant did not, while at 2 μg/ml vancomycin the mutant grew significantly more slowly than wt (Fig. 1A). Previous studies showed that tRNA^Other^ can be aminoacylated with lysine *in vitro*, albeit poorly [17], prompting us to test for tRNA^Other^-dependent lysylation of membrane lipids which could be expected to influence antibiotic resistance [5], [20], [21]. No differences were observed in either total lipid synthesis or the degree of membrane lysylation in wild-type (wt) relative to *B. cereus* Δ*tRNA^Other^*, indicating that tRNA^Other^ does not function in tRNA-dependent lipid modification (data not shown). To investigate if accumulation of suppressor mutations contributed to the observed phenotypes, stationary phase wt and Δ*tRNA^Other^* cells grown in the presence of 2 μg/ml vancomycin were used to inoculate fresh antibiotic-containing media. These re-inoculated cultures produced growth curves indistinguishable from the original cultures, indicating that the deletion of tRNA^Other^ was responsible for the observed change in vancomycin resistance.

**Table 1:** Phenotypes^1^ of *B. cereus* Δ*tRNA^Other^* relative to wt determined by BIOLOG phenotypic array analyses.

Resistances Lost (intensity)	Mode of Action/Target
Benzethonium Cl (-148)	Membrane, detergent, cationic
Chelerythrine (-201)	Protein kinase C
Dodecyltrimethyl NH_4_Br (-155)	Membrane, detergent, cationic
Domiphen bromide (-166)	Membrane, detergent, cationic, fungiside
Iodonitro Tetrazolium (-312)	Respiration
Lauryl sulfobetaine (-126)	Membrane, detergent, zwitterionic
Nafcillin (-105)	Cell wall biosynthesis
Niaproof (-160)	Membrane, detergent, anionic
Novobiocin (-141)	DNA topoisomerase
Pentachlorophenol (-146)	Respiration, ionophore, H^+^
Puromycin (-158)	Protein synthesis, 50S ribosomal subunit
Sanguinarine (-124)	ATPase, Na^+^/K^+^ and Mg^2+^
Tetrazolium Violet (-441)	Respiration
Trifluoperazine (-230)	Cell cycle modulation, DNA synthesis
Tylosin (-108)	Protein synthesis, 50S ribosomal subunit, macrolide
Vancomycin (-270)	Cell wall biosynthesis

1 Only a subset of phenotype changes is shown corresponding to intensity losses above an arbitrary threshold of 100 units or greater. Intensity corresponds to the area under the curve divided by number of reads. The array was conducted in duplicate after incubation of the strains at 37°C for 24 h.

The bactericidal activity of antibiotics such as vancomycin has previously been linked to iron-induced oxidative stress to [22], [23], and a possible role for iron in regulating tRNA^Other^ (see below), prompted us to investigate changes in antibiotic resistance upon addition of the iron chelator dipyridyl (DIP). DIP partially restored wt-like growth rates to *B. cereus* Δ*tRNA^Other^* grown in the presence of vancomycin (Fig. 1A), suggesting that tRNA^Other^ may be involved in regulating aspects of the oxidative stress response. The sensitivity of wt and *B. cereus* Δ*tRNA^Other^* to direct oxidative stress was tested by comparing growth of these strains in media containing H_2_O_2_. Growth of *B. cereus* Δ*tRNA^Other^* was abolished with 1 mM H_2_O_2_, while the wt strain continued to grow (data not shown). When exposed to 400 μM H_2_O_2_, *B. cereus* Δ*tRNA^Other^* growth was reduced relative to wt (Fig. 1B). The growth defect observed for *B. cereus* Δ*tRNA^Other^* in response to H_2_O_2_ closely resembles the growth defect observed in response to vancomycin (Fig. 1A).

The susceptibility of microbes to antibiotics due to oxidative stress can be alleviated by increased nitric oxide (NO) production [24]. NO levels were significantly lower in *B. cereus* Δ*tRNA^Other^* than wt, indicating that NO production is regulated by tRNA^Other^ (Fig. 1C). However, as addition of DIP reduced NO levels in both strains, these data indicate that NO levels are also regulated independently of tRNA^Other^ in *B. cereus.*

**Figure 2:**
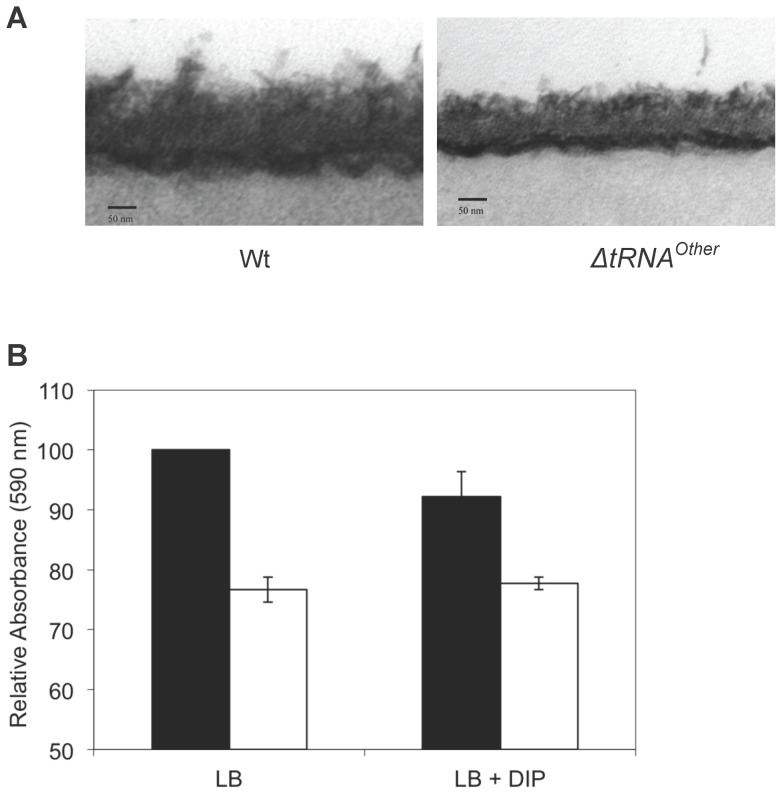
Morphology of wt and Δ*tRNA^Other^ B. cereus.* **A**, TEM of wt and Δ*tRNA^Other^ B. cereus* cell walls. Average cell wall thickness: wt, 60.4±4.6 nm; Δ*tRNA^Other^*, 45.0±3.9 nm. **B**, Biofilm formation. Wt (black bars) and Δ*tRNA^Other^* (white bars) strains were grown under high and low (+DIP) iron conditions. Data was normalized to wt *B. cereus* grown in LB.

Several reports have also linked reduced resistance to antibiotics and oxidative stress to decreased cell wall thickness [25], [26]. Transmission electron microscopy revealed that *B. cereus* Δ*tRNA^Other^* did not show significant differences in cell shape or elongation but had a 30% thinner cell wall than wt (Fig. 2A), indicating that tRNA^Other^-dependent morphological changes may also contribute to antibiotic resistance. Monitoring of surface growth in 96 well plates revealed additional changes after deletion of tRNA^Other^, biofilm formation by *B. cereus* Δ*tRNA^Other^* being significantly lower than wt, regardless of whether or not DIP was included in the growth medium (Fig. 2B).

### tRNA^Other^–dependent changes in transcription

**Figure 3:**
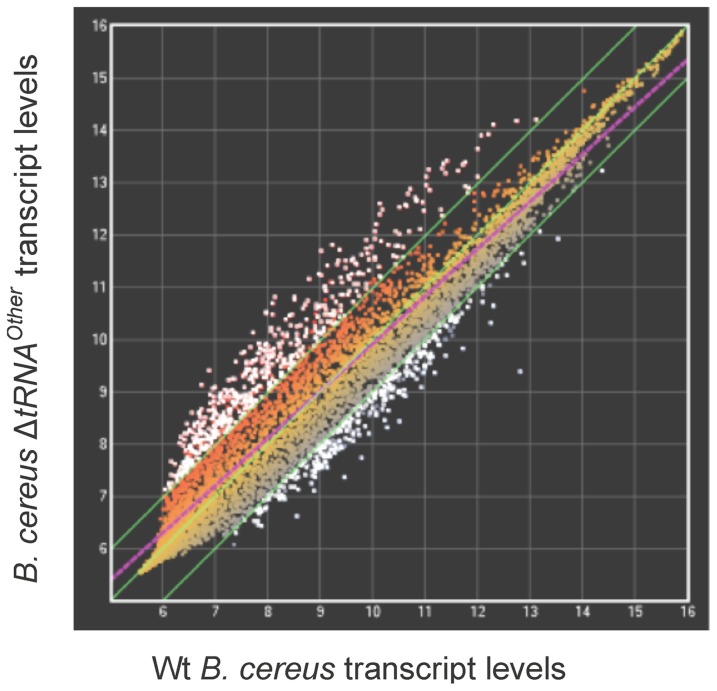
Transcript profiles of wt and *B. cereus* Δ*tRNA^Other^*. Transcript level data is presented as the average of two microarrays. Each dot represents the transcript level for one gene. The three green lines, from top left to bottom right, indicate 2-fold higher, equal, and 2-fold lower transcript levels for Δ*tRNA^Other^* relative to wt *B. cereus*. White dots represent transcripts with a significant 2-fold change or greater.

**Figure 4:**
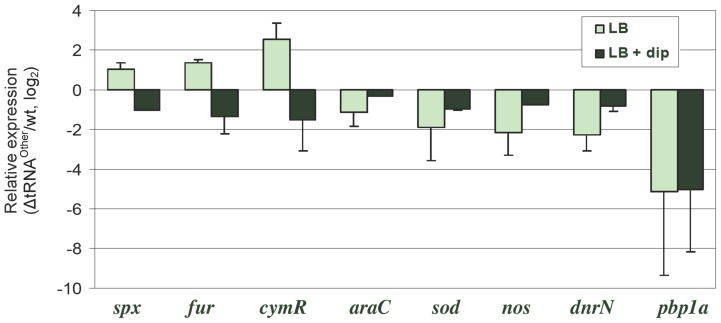
Changes in gene expression due to the deletion of tRNA^Other^. Expression of genes in *B. cereus* Δ*tRNA^Other^* reletive to wt grown in rich media (light green) and iron depleted media (dark green) was determined by qRT-PCR. Data presented in log_2_.

Transcriptional microarrays were used to explore the effects of tRNA^Other^ on gene expression. Significant differences in transcript levels between the wt and Δ*tRNA^Other^* strains were observed for 773 genes during exponential growth, (2-fold change or higher, p-value ≤0.05; Fig. 3). The largest difference observed on deletion of tRNA^Other^ was a 10.8-fold reduction in the level of the transcript encoding the putative multimodular transpeptidase-transglycosylase PBP 1a, which functions in peptidoglycan biosynthesis [27]. 2- to 4-fold changes in transcript levels were observed for a number of other genes, including the transcriptional regulators *fur*, *spx*, *cymR*, *araC*, and *dnrN*, and many members of their respective regulons. Quantitative reverse transcription-PCR (qRT-PCR) was performed to further investigate transcript level changes detected by transcriptional microarray analysis. As the expression of tRNA^Other^ itself is repressed by iron (see below), RNA was prepared from strains grown in LB with addition of the iron chelator DIP. For cells grown in LB without DIP, transcript level changes for many genes were mostly comparable between the qRT-PCR and microarray analyses. The transcript level changes for three genes contradicted those determined by transcriptional microarray analyses. These three genes, s*px*, *cymR-*homologue, and *fur*, encode transcriptional regulators involved in the oxidative stress response. While the transcriptional microarray results indicated a decrease in transcript levels for these three genes in the tRNA^Other^ deletion strain relative to wt, the levels determined by qRT-PCR from cells grown in LB without DIP were higher in *B. cereus* Δ*tRNA^Other^* when compared to wt. However, when cells were grown in the presence of DIP, the transcript level changes for these genes were comparable to the changes observed in the transcriptional microarray analysis: transcript levels from all three genes were lower in Δ*tRNA^Other^* than in the wt strain (Table 2). These somewhat contradictory data likely result from differences in fluctuating iron concentrations in water used to prepare LB medium batches used for the transcriptional microarray, whereas the qRT-PCR experiments were performed under more controlled iron regimes. Consequently, further analysis of the possible role of tRNA^Other^ in regulation was confined to analysis of qRT-PCR acquired data (Fig. 4). Under low iron conditions (250 μM DIP), *spx*, *cymR*-homologue, and *fur* transcript levels were significantly decreased in the Δ*tRNA^Other^* strain relative to wt, while an increase in these transcript levels was observed under higher iron conditions (rich medium, no DIP). These results indicate that the presence of tRNA^Other^ is required for a significant reduction in the transcript levels of these genes when cells are grown under conditions with low iron concentration. Some of the genes for which the qRT-PCR and microarray analyses were comparable are also involved in the oxidative stress response, such as those encoding superoxide dismutase [Fe/Mn] (SOD), nitric oxide synthase (NOS), and nitric oxide dependent regulator (DnrN). *sod* transcript levels were 2.0- to 3.7-fold lower in the Δ*tRNA^Other^* strain than the wt with or without DIP (Table 2). Transcript levels for *pbp1a* were comparable between microarray and qRT-PCR data for Δ*tRNA^Other^* relative to wt grown with or without DIP, indicating that deletion of tRNA^Other^ is a major factor contributing to the decrease in *pbp1a* transcript levels.

**Table 2:** Changes in transcript levels between wt and *B. cereus* Δ*tRNA^Other^* during growth under moderate and low iron conditions.

	Predicted	Microarray	qRT-PCR			
Gene #	Gene Product	Δ/wt*	Δ /wt	Δ +DIP/wt+DIP^†^	wt+DIP/wt	Δ +DIP/Δ
BC1188	Spx	−4.2	2.0	−2.0	1.6	−2.0
BC2773	CymR-homologue	−2.6	4.7	−2.8	−1.6	−11
BC4901	Fur	−2.2	2.5	−2.5	−2.2	−17
BC2632	AraC	−2.6	−2.2	−1.2	−1.8	1.3
BC5445	SOD [Mn]	−2.7	−3.7	−2.0	−5.1	−3.6
BC5444	NOS	−1.6	−5.1	−1.7	−6.3	−2.7
BC2137	DnrN	−3.4	−4.9	−1.7	−4.6	−1.7
BC1550	PBP 1a	−10.8	−36	−32	−1.3	−2.6
BC2458	Thioesterase ‡	1.1	1.1	1.1	1.0	1.0
BC1232	Anthrinillate synthase ‡	1.0	−1.1	1.0	−1.1	1.0

* Δ, *B. cereus* Δ*tRNA^Other^*; wt, wild-type *B. cereus* ATCC14579.

‡ Negative control transcript levels were determined with a standard curve.

### tRNA^Other^ transcript mapping

**Figure 5:**
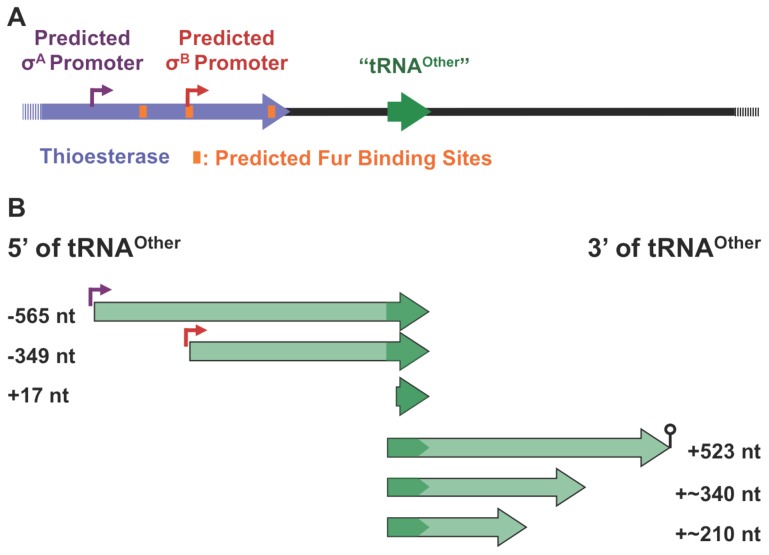
Mapping of tRNA^Other^. **A**, Putative promoter and Fur binding sites (DBTBS [28]). Thioesterase indicates the ORF immediately 5′ of tRNA^Other^. **B**, 5′ and 3′ mapping by RACE. Circle with a vertical line at the bottom indicates an intrinsic terminator. Numbers indicate 5′ and 3′ nt relative to the originally predicted 5′-and 3′- ends of *tRNA^Other^*.

Since neither this nor previous studies suggested roles for tRNA^Other^ consistent with known cellular functions of tRNAs and pseudo-tRNAs, the synthesis and maturation of tRNA^Other^ was investigated by 5′-rapid amplification of cDNA ends (5′-RACE). No products were visible from the initial PCR other than primer dimers (at about 65 bp), suggesting a low abundance of tRNA^Other^ in the total RNA fraction extracted from *B. cereus*. After a second round of PCR with nested primers, several products were clearly visible and these were cloned and sequenced. The longest cDNA fragment, which starts 565 nt upstream of the original putative tRNA^Other^ 5′ end, corresponds to a site nine nucleotides downstream of a predicted σ^A^ promoter (Fig. 5) [28]. The sequence beginning 349 nt upstream of the original tRNA^Other^ 5′ end corresponds to a site 16 nt downstream of a predicted σ^B^ promoter [28]. The third 5′-RACE product, which appeared in the majority of the cloned sequences, was 17 nt shorter than the original tRNA^Other^ size prediction. 3′-RACE products were not as distinct as those for 5′-RACE. A smear of DNA product could be seen after the first PCR, ranging from approximately 125 to 600 bp. The second PCR, using nested primers, showed more defined DNA bands, yet still within a large range of sizes. Sequencing of the largest bands (∼1.75 and 3 kb) identified 23S and 16S rRNA rather than sequences specific to tRNA^Other^. Of the products consistent with a possible 3′-end, only a few had sequences with similar 3′-ends. The majority of these sequences end within a predicted intrinsic transcriptional terminator 523 nt downstream of the original tRNA^Other^ sequence (Fig. 5). Other sequences ended approximately 340 and 210 nt downstream of the original tRNA^Other^ sequence, but are variable in their exact 3′-end site and do not have any predicted transcriptional terminators. Taken together these findings indicate that tRNA^Other^ is transcribed as part of a larger RNA.

### Transcription of tRNA^Other^ is regulated by iron

**Figure 6:**
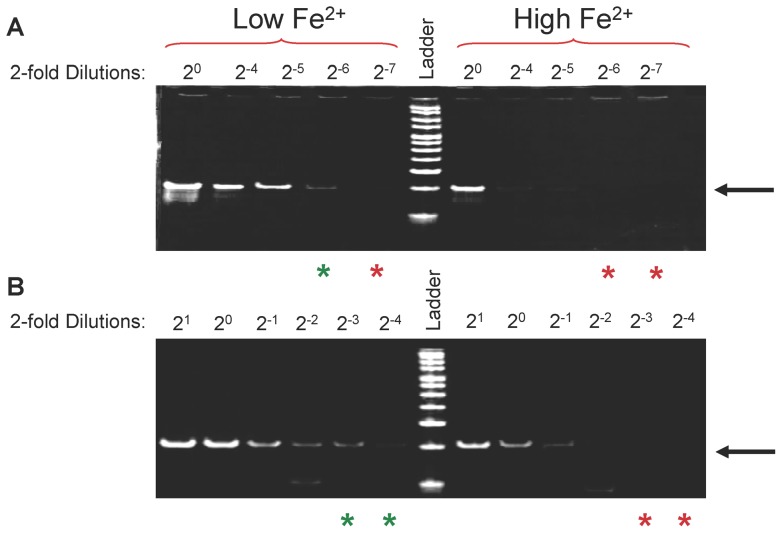
Effect of extracellular iron on tRNA^Other^ transcription. Low iron concentration was achieved by addition of the iron chelator DIP (250 μM). High iron concentration was achieved by the addition of FeSO_4_ (250 μM). Green and red asterisks indicate presence and absence of a PCR signal, respectively. DNA marker is HyperLadder V (Bioline). PCR of 2-fold dilutions of tRNA^Other^ cDNA from cells grown for 30 (**A**) and 120 min (**B**), in LB with low (Lanes to the left of the DNA marker) or high (Lanes to the right of the DNA marker) iron concentrations. Arrows indicate RT-PCR product for tRNA^Other^ at 74 bp.

Deletion of tRNA^Other^ led to widespread changes in the expression of genes from several stress-response regulons, including the ferric uptake regulator (Fur). Utilizing DBTBS (database of transcriptional regulation in Bacillus subtilis; http://dbtbs.hgc.jp/), Fur binding sites were predicted upstream of the tRNA^Other^ encoding region and overlapping a putative σ^B^ promoter (Fig. 5) [28]. Regulation by Fur was investigated by comparing tRNA^Other^ levels during growth with excess or depleted iron. Since tRNA^Other^ is predicted to have a stable secondary structure, PCR of cDNA serial dilutions, rather than quantitative PCR, was performed to determine relative concentrations. As a control, *fur* transcripts were first monitored and, as expected, elevated iron increased their levels ∼2-fold after 30 min of growth (data not shown). Under the same conditions as those used to monitor *fur*, tRNA^Other^ levels decreased ∼2-fold after 30 min and ∼4-fold after 120 min growth in the presence of elevated iron levels (Figs. 6A and 6B), supporting the prediction that iron can regulate tRNA^Other^ transcription.

### Prediction of mRNA targets for tRNA^Other^-containing transcripts

**Figure 7:**
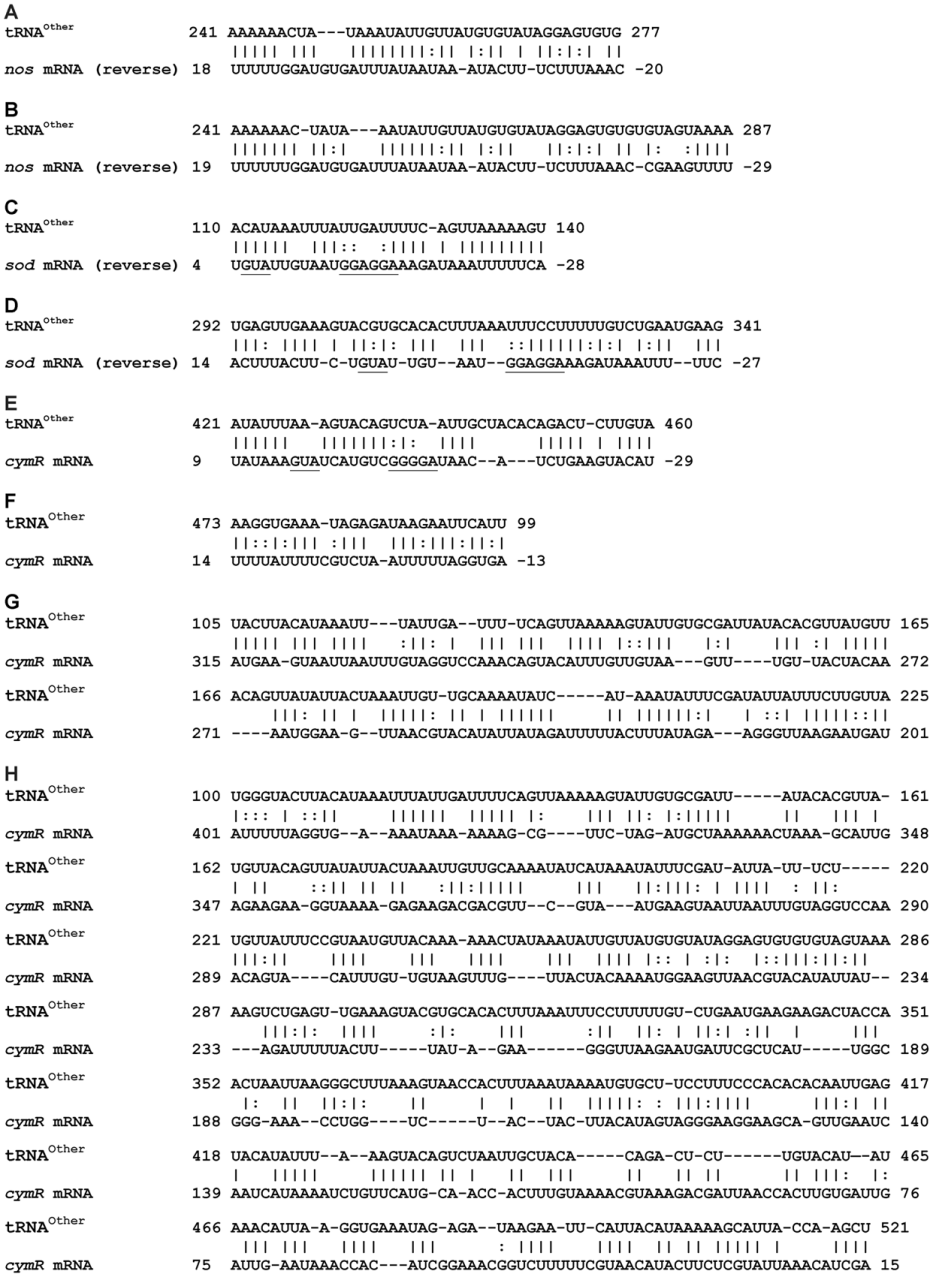
Predicted interactions between tRNA^Other^-containing transcripts and putative mRNA targets. Target prediction parameters for *nos* (**A** and **B**) and *sod* (**C** and **D**) included terminator removal, a hybridization seed of 9, G:U basepairs included, and alignment score determined with a P-value set to 0.05 (**A** and **C**) or thermodynamic energy (**B** and **D**). Vertical lines (|) indicate a Watson-Crick base pair, and dots (**:**) indicate a G-U wobble base pair. The numbered nucleotide positions are relative to the start and stop codons for *sod* and *nos* mRNAs, respectively. Target prediction parameters for *cymR* included terminator removal, a hybridization seed of 8, and G:U basepairs included. Alignment was focused on the start codon (**E**), the stop codon (**F**), or the coding sequence (**G** and **H**). Alignment score was determined with a P-value set to 0.01 (**C**) or thermodynamic energy (**E**, **F** and **H**). Vertical lines (|) indicate a Watson-Crick base pair, and dots (**:**) indicate a G-U wobble base pair. The numbered nucleotide positions are relative to the start and stop codons for *cymR* mRNA. Ribosome-binding sites and start codons are underlined (note that *sod* and *cymR* homologue sequences are written 3′ to 5′. Targets were predicted using TargetRNA and sRNATarget.

Recent studies have revealed numerous bacterial RNAs that utilize a variety of mechanisms to regulate virulence and other cellular stress responses [29], [30]. TargetRNA and sRNATarget software programs were used to identify mRNA targets for the tRNA^Other^-containing transcripts predicted by 5′- and 3′-RACE [31], [32]. Using RNA sequences spanning from the shortest observed 5′-end to the predicted intrinsic transcriptional terminator, both programs predicted *cymR*-homologue, *nos* and *sod* mRNAs as targets, all of which were implicated in our transcriptome analysis (Table 2). The predicted site of interaction between tRNA^Other^-containing transcripts and the *cymR-*homologue and *sod* mRNAs is focused around the ribosome binding site and translational start site, a common strategy employed by sRNAs that act by modulating ribosome recruitment [33]. The *cymR-*homologue also showed additional complementarities with tRNA^Other^-containing transcripts extending for several hundred nucleotides from just after the predicted translation start site (Fig. 7). Target complementarity with *nos* centered instead around the predicted stop codon, suggesting that in this case tRNA^Other^-containing transcripts might act by regulating mRNA stability. Taken together, these data predict that tRNA^Other^-containing transcripts may have a direct regulatory effect on the expression of at least three genes expected to help increase antibiotic resistance, perhaps as part of a broader oxidative stress response regulated by iron. Further support for direct interactions between tRNA^Other^-containing transcripts and their putative targets requires expression in trans of the predicted mature form(s) of the transcripts in both wt and Δ*tRNA^Other^* mutant strains and complementation of the corresponding phenotypes; however all efforts to date to achieve this goal using a variety of both plasmid-borne and chromosomal expression systems have proved unsuccessful (data not shown).

## Discussion

### tRNA^Other^ is a tRNA-like element within a regulatory RNA

tRNAs play an essential role in the translation of the genetic code, and recent studies have now shown that tRNAs also play numerous additional roles outside mRNA translation [5]. These functions vary greatly from the biosynthesis of amino acids such as selenocysteine [34] to the regulation of global transcript profiles as in the stringent response [35]. Recent studies have also started to show that the inherently stable secondary and tertiary structure of tRNAs may provide a suitable framework for regulatory small RNAs, whether as whole or cleaved tRNAs, as exemplified by tRNA regulatory fragments, or tRFs [36]–[41]. The data presented here suggest that tRNA^Other^ may play a similar role by providing a structural element within a larger regulatory RNA. Whether the various tRNA^Other^-containing transcripts detected by RACE are distinct variants resulting from specific processing steps, or are instead degradation intermediates, is currently unclear.

Comparisons to other closely related species revealed that several genomes of the *B. cereus* group, but as of yet no other organisms, contain sequences with extensive similarity to the tRNA^Other^-containing transcripts described here. The highest similarity is seen in the genome of *Bacillus thuringiensis* BMB171, which contains a continuous region with 98% nucleotide identity to the 5′ 565 nt and 3′ 523 nt flanking regions (Fig. 5b) and 100% identity to tRNA^Other^. The *B. cereus* strains B4264 and G9842 also show extensive sequence similarity, although not in one single region of their genomes; B4264 has an insertion (133 bp) in the 3′ end of tRNA^Other^ while *B. cereus* G9842 has sequence similarity to the 3′ end of the thioesterase gene and to the 523 nt 3′ flanking tRNA^Other^ but at a distal location in the genome. The conservation in other Bacilli of sequences similar to the tRNA^Other^-containing transcripts described here may indicate a similar role for such RNAs in these organisms.

### Regulation of tRNA^Other^

The Fur binding sites present on and around the σ^B^ promoter upstream of the tRNA^Other^ coding region are commonly observed for Fur regulated genes [42], consistent with our observation that tRNA^Other^ expression is regulated by iron. Under high levels of ferrous iron (Fe^2+^), Fur binds excess iron and is activated for DNA binding. Fur usually acts as a repressor to reduce transcription of genes, as is the case here for tRNA^Other^. Fur-regulated sRNAs have been shown to play key regulatory roles in iron homeostasis in a variety of bacteria, [43]–[48]. Although tRNA^Other^ has almost no sequence homology to any of the other known Fur-regulated sRNAs, little to no sequence homology or similarity in RNA length exists between most of these sRNAs [46]. Fur-regulated sRNAs have also been shown to function in processes other than iron homeostasis. For example, Fur-regulated RNAs were found to affect acid resistance in *Shigella flexerni* [48] and motility, chemotaxis, and biofilm formation in *Vibrio cholerae* [49]. Iron-regulation of tRNA^Other^ has some comparable roles in the cell, leading to changes in antibiotic resistance, biofilm persistence and cell wall morphology. These phenotypes may in part be explained by the substantial changes seen in expression of *pbp1a*, which encodes a transglycosylase-transpeptidase involved in cell wall biosynthesis [25], [27], changes in which have been shown to effect vancomycin resistance [26]. The role of Pbp1a in modulating the oxidative stress response and antibiotic resistance in *B. cereus*, and how it is regulated by tRNA^Other^, could not be assessed directly as we were unable to disrupt the *pbp1a* gene, suggesting it may be essential (data not shown). Nevertheless, the concomitant changes in *pbp1a* expression and cell wall morphology following deletion of tRNA^Other^ suggest a possible mechanism to modulate resistance to some antibiotics.

### Modulation of antibiotic resistance by tRNA^Other^

**Figure 8:**
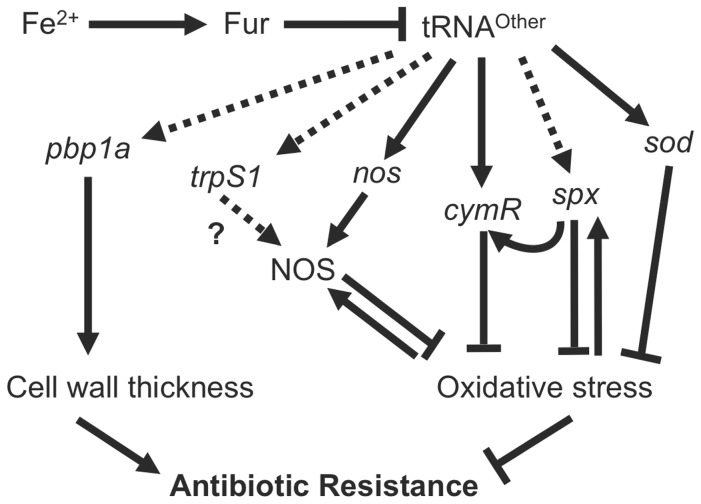
Proposed role for tRNA^Other^ in *B. cereus* antibiotic resistance. Low levels of ferrous iron (Fe^2+^) limit DNA binding by Fur, which de-represses expression of tRNA^Other^. tRNA^Other^ induces expression of *pbp1a*, *trpS1*, *nos*, *cymR*, *spx*, and *sod* by undetermined mechanisms. An increase in *pbp1a* expression leads to an increase in cell wall thickness, which can reduce susceptibility to certain antibiotics. TrpRS1 interacts with and induces activity of NOS, which, together with SOD, reduces endogenous oxidative stress. CymR and Spx are both involved in regulation of gene expression in the response to, and in order to combat, oxidative stress.

Recent studies indicate that bactericidal antibiotics can kill bacteria by inducing intracellular oxidative stress [22], [50]. tRNA^Other^ affects transcription of the genes encoding TrpRS1 [18], NOS, DnrN, SOD, CymR-homolog, Spx, and Fur (this study), all of which could potentially modulate antibiotic resistance via changes in the oxidative stress response. For example, the derepression of *trps1* is expected to increase the level of TrpRS1, which may then interact with and induce NO production by NOS [51]. Bacterial NOS produces intracellular nitric oxide in quantities that protect the cell from oxidative stress [24], [52]. DnrN is an NO-responsive protein thought to protect the cell from NO-induced damage by Fe-S cluster repair and/or reduction of intracellular NO concentration [53]–[55]. SOD destroys highly reactive superoxides, converting them to the less reactive species H_2_O_2_ [52], [56]. The CymR-homologue is a member of the Spx regulon, and both transcriptional regulators are involved in regulating gene expression in the response to oxidative stress. CymR regulates the biosynthesis of cysteine, which acts as a cellular reductant in *B. cereus* to convert Fe^3+^ to the redox active Fe^2+^. Taken together, our data suggest that tRNA^Other^-containing transcripts have the potential to impact the expression of numerous genes responsible for reducing intracellular oxidative stress, and in so doing globally modulate the resistance of *B. cereus* to bactericidal antibiotics such as vancomycin (Fig. 8).

## Materials and Methods

### Bacterial strains, growth conditions, and BIOLOG analysis

Wt *B. cereus* ATCC14597 and the previously described isogenic Δ*tRNA^Other^* deletion strain [18] were grown in LB broth with vigorous shaking at 37°C. Growth was monitored by measuring absorbance at 600 nm. Overnight cultures were diluted into 50 ml fresh, pre-warmed LB to reach mid-exponential phase twice prior to inoculation of larger batch cultures. When appropriate, vancomycin (100, 50, 10, 5, or 2 μg/ml), H_2_O_2_ (2, 1, 0.5, 0.4, 0.2, or 0.1 mM), DIP (250 μM), and FeSO_4_ (250 μM) were added to the culture media. BIOLOG analyses were performed by comparing the changes in cell respiration of wt and *B. cereus* Δ*tRNA^Other^* in ∼1,900 different culture conditions. Each condition was repeated twice and fitted into a threshold of confidence for each strain. The BIOLOG analysis was performed by Biolog, Inc (Hayward, CA).

### Total RNA extraction and purification

For transcriptional microarray analysis and subsequent confirmation of transcript levels via qRT-PCR, total RNA was stabilized with RNAprotect (QIAGEN) and isolated with RNaeasy mini kit (QIAGEN) according to the manufacturer's instructions except as noted. Briefly, 5 ml of cells from the mid-exponential growth phase were pelleted at 5,000 x g, 4°C, resuspended in 1 ml RNAprotect, incubated at room temperature for 5 min, re-pelleted, and stored at −80°C. Cells were suspended in lysis buffer (30 mM Tris-HCl, pH 8.0, 1 mM EDTA, 15 mg/ml lysozyme) with 30 mg acid washed beads and vortexed in a multivortexer for 10 min at room temperature. Buffer RLT (700 μl) was added and cell suspension was vortexed for an additional 5 min before continuing with the manufacturer's instructions. DNA digestion was performed with TURBO™ DNase (Ambion) followed by a control PCR to check for residual contamination of the isolated RNA with DNA. RNA quality and concentration were determined using an ND-1000 spectrophotometer (NanoDrop Technologies, Inc.) and visualized by formaldehyde-agarose gel electrophoresis.

### Transcriptional microarrays

Double-stranded (ds) cDNA for transcriptional microarrays was prepared according to Roche NimbleGen, Inc. instructions using the SuperScript™ double-stranded cDNA synthesis kit (Invitrogen). Total RNA was isolated as described above and pooled from cultures grown in triplicate prior to ds cDNA production. The Roche NimbleGen, Inc. *Bacillus cereus* ATCC 14579-specific bacterial gene expression array includes 5,255 protein-coding genes from both chromosomal and plasmid DNA, accession numbers NC_004721 and NC_004722, respectively. Each target ORF is represented by four replicates of 18 unique 60-mer probes arranged in a 385K format. Transcriptional microarrays were performed in duplicate for mid-exponential phase wt and *B. cereus* Δ*tRNA^Other^*. Data were normalized by the quantile method and analyzed with the RMA algorithm using ArrayStar® v2.1 software [57]–[58]. Significance was determined by a student's *t*-test with the Benjamini and Hochberg False Discovery Rate (FDR) correction resulting in a *P* value ≤0.05 [59]. The description and analysis of these arrays will be deposited in NCBI Gene Expression Omnibus (GEO) under platform accession number GPL15435 (http://www.ncbi.nlm.nih.gov/geo/query/acc.cgi?acc=GPL15435).

### Quantitative RT-PCR

All primers and probes were designed using GenScript Real-time PCR (TaqMan) Primer Design (https://www.genscript.com/ssl-bin/app/primer) to produce a single PCR product of 75–250 bp, based on amplification of wt *B. cereus* genomic DNA. Reverse transcription was performed using SuperScript II reverse transcriptase (Invitrogen) according to the manufacturer's instructions with gene-specific 3′-primers (1 μM) and total RNA (40 ng or 400 ng). Quantitative PCR was performed using iQTM SYBR® Green Supermix with 1 μl cDNA and gene specific primers (300 nM). The Opticon2 DNA Engine (BioRad) was used with an initial 3 min incubation at 94°C, 40 cycles of 94°C for 30 sec, 60°C for 30 sec, and 72°C for 30 sec followed by a plate read. Melting curves (50–95°C) were performed for each PCR to confirm the presence of the correct amplicon and absence of primer dimers. For each reaction, a negative control was performed without reverse transcription. RNA preparation and qRT-PCR was repeated three times and transcript level changes were averaged. Transcript level changes were determined by the Pfaffl method [60]. BC2458 and BC1232 (encoding thioesterase and anthrinillate synthase, respectively) were chosen to serve as reference genes due to the lack of any transcript level change between wt and Δ*tRNA^Other^* grown with or without DIP, as shown in both the transcriptional microarray analysis and qRT-PCR. Reference gene transcript levels were quantified by comparing to data collected for genomic DNA standards for qRT-PCR.

### Measurement of iron-dependent gene expression

*B. cereus* wt and Δ*tRNA^Other^* were grown overnight in 5 ml LB at 37°C with shaking at 250 rpm, and these cultures used to inoculate 25 ml of pre-warmed LB. After incubation for 1.5 h at 37°C, these cultures were used to inoculate 25 ml of pre-warmed LB to an OD_600_ of 0.1. The cultures were incubated for an additional 1.5 h at 37°C and subsequently used to inoculate 100 ml LB plus appropriate antibiotics and/or DIP to an OD_600_ of 0.05 or 0.1. When appropriate, DIP (250 μM) or FeSO_4_ (250 μM) were added to growth media. RNA was extracted and purified as described and 400 ng used for reverse transcription with 1 pmol 3′ primer and SuperScript II reverse transcriptase (Invitrogen) according to manufacturer's instructions, except annealing and reverse transcription temperatures were altered for tRNA^Other^, 80°C for 3 min then 50°C for 15 min, and 50°C for 35 min then 42°C for 20 min, respectively. PCR was performed with 300 nM of each primer and Taq DNA polymerase (Invitrogen) for 35 cycles of 95°C for 45 sec, 55°C for 45 sec, and 72°C for 45 sec.

### Intracellular nitric oxide detection

50 to 100 ml of mid exponential phase cells were pelleted at 4,000 x g for 7 min at 4°C for both wt and *B. cereus* Δ*tRNA^Other^*. Cells were washed three times in Hank's Buffer (0.137 M NaCl, 5.4 mM KCl, 0.25 mM Na_2_HPO_4_, 0.44 mM KH_2_PO_4_, 1.3 mM CaCl_2_, 1.0 mM MgSO_4_, 4.2 mM NaHCO_3_, 1 g/L glucose, without phenol red). Cells were resuspended in Hank's Buffer at room temperature to obtain a final volume of 1 ml and an OD_600_  = 8.0. The NO probe 1,2-diaminoanthraquinone (DAA) was added to the resuspended cultures (50 μg/ml) and incubated in the dark at 37°C with gentle agitation for 1 h. Cells were washed three times in Hank's Buffer at room temperature, resuspending each time with gentle pipetting. Cells were resuspended in 1 ml Hank's Buffer and stored briefly at room temperature in the dark until visualized via fluorescence microscopy with a motorized, inverted epi-fluorescence microscope (Axio Observer D1, Zeiss) using a 300 W Xenon Arc bulb (Lambda DG-4, Sutter Instrument Company) and a Cascade II 512 EMCCD camera from Photometrics. The software Metamorph “Premier” (Molecular Devices) was used to drive the microscope equipment and perform image analyses [61]. Phase contrast and fluorescence (filter set 49005, Chroma Technology Corporation) images were acquired with a 100X objective (N.A.  = 1.4) to delineate total cells and measure their fluorescence intensities, respectively. As a control for autofluorescence, cells were prepared as described, except without the addition of DAA. Average individual cell fluorescence was assessed following background correction (subtraction of the dark noise and autofluorescence).

### Biofilm formation assay

Wt and *B. cereus* Δ*tRNA^Other^* were grown to stationary phase (∼18 h) in 5 ml of LB at 37°C with shaking, then diluted to an OD_600_ of 0.1 in fresh LB containing 500 μM DIP. 200 μl aliquots of diluted culture were placed in each of the 350 μl wells of a 96-well microtiter plate. After incubation at 37°C for 24 and 48 h, wells were rinsed 5 times with phosphate buffered saline (PBS), stained with 250 μl crystal violet for 15 min, then rinsed another 5 times with PBS. The crystal violet retained by the biofilm was dissolved with 250 μl of 100% ethanol, and absorbance was measured at 590 nm. Three replicates were performed.

### Transmission electron microscopy

Wt and *B. cereus* Δ*tRNA^Other^* strains were grown as described above, except cells were grown to OD_600_  = 0.3. Cells were then harvested and fixed overnight in 3% glutaraldehyde, 0.1 M sucrose, and 0.1 M phosphate buffer, pH 7.4 at 4°C. Cells were then washed three times (0.1 M sucrose and 0.1 M phosphate buffer, pH 7.4) and post-fixed in 1% osmium tetroxide (Ted Pella, Inc., Redding, CA) for 1 h at room temperature. Samples were *en bloc* stained in 1% uranyl acetate and dehydrated in a graded series of ethanol and propylene oxide before embedding in Eponate 12 resin (Ted Pella, Inc., Redding, CA). Thin sections were cut on a Leica EM UC6 Ultramicrotome (Leica Microsystems, Exton PA), stained with lead citrate and observed on an FEI Tecnai G2 Spirit transmission electron microscope (FEI, Hillsboro, OR). Secondary fixation, staining and microscopy were performed at the Ohio State University Campus Microscopy and Imaging Facility. Cell wall thickness was measured using Adobe Photoshop CS3 Extended version 10.0.1. To obtain an average cell wall thickness for each cell, measurements were taken every 50 nm along the long sides of the rod-shaped cell at a perpendicular angle to its length. Cell poles and visible division sites were avoided in the measurements as cell wall thickness in these areas was variable relative to the long sides of the cell.

### 5′ RACE

Preliminary mapping of the 5′ end of sRNA^Other^ was performed using various sets of primers for RT-PCR, each one containing the same 3′ primer and a variable 5′ primer. RT-PCR was performed as described above, except the 5′ primer varied in each reaction. Mapping of the 5′ end was performed using a 3′/5′ RACE kit (Rapid Amplification of cDNA Ends, Roche). cDNA was synthesized from total RNA (isolated from cells grown with or without DIP) with primer BCother3 (5′-TGGAGTGGATGGCGGGAATT-3′, 0.625 μM) and Transcriptor Reverse Transcriptase in reaction buffer containing dNTP mix (1 mM each), 50 mM Tris-HCl, 8 mM MgCl_2_, 30 mM KCl, 5 mM DTT, pH 8.5, and incubated at 55°C for 60 min. cDNA purification was performed with the High Pure PCR Purification kit (Roche). The first PCR was performed using the Expand High Fidelity^PLUS^ PCR System (Roche) with a nested 3′ primer, BCother3 nest1 (5′-GAATTGAACCCACATCAGAGG-3′, 0.25 μM), and Oligo dT-anchor primer (5′-GACCACGCGTATCGATGTCGACTTTTTTTTTTTTTTTTV-3′, where V  =  A, C, or G, 0.25 μM). A second PCR protocol was performed using 1 μl of products of the first PCR as the template, and the the 3′ nested primer BCother3 nest2 (5′-GTTCTGGAGGCCCCTGTTTT-3′) and the 5′ PCR anchor primer provided by Roche. PCR products were visualized after running an aliquot from each reaction on a 2% agarose gel. Aliquots of both the first and second PCRs, and DNA bands visible by agarose gel electrophoresis, were cloned into the Zero Blunt TOPO-cloning vector (Invitrogen) and subsequently sequenced.

### 3′ RACE

Mapping of the sRNA^Other^ 3′ end was performed using the 3′/5′ RACE kit (Rapid Amplification of cDNA Ends, Roche). Total RNA was treated with *E. coli* poly (A) polymerase (NEB) (4 μg RNA, 50 mM Tris-HCl, 250 mM NaCl, 10 mM MgCl_2_, 1 mM ATP, pH 7.9, incubated for 10 min at 37°C) followed by two phenol/chloroform extractions and ethanol precipitation. cDNA was synthesized from 3′-poly (A) tailed total RNA (2 μg) with Oligo dT-anchor primer and Transcriptor Reverse Transcriptase. The first PCR was performed using the Expand High Fidelity^PLUS^ PCR System with BCother5 nest1 primer (5′-CCTCTGATGTGGGTTCAATTC-3′, 0.25 μM) and PCR anchor primer. Because only a light smear of DNA was visible by agarose gel electrophoresis of the first PCR products, a second PCR protocol was performed using 1 μl of the first PCR as the template. All conditions were the same as for the first PCR, except the annealing temperature was raised from 60 to 65°C, and the 5′ primer used was BCother5 nest3 (5′-CAATTCCCGCCATCCACTTATACTTG-3′). Aliquots of the second PCRs were cloned with the Zero Blunt TOPO-cloning vector (Invitrogen). Sections of the DNA smear visible by agarose gel electrophoresis were purified using the QIAGEN Gel Purification kit and subsequently cloned with the Zero Blunt TOPO-cloning vector and sequenced.
